# Dietary homogenization and spatial distributions of carbon, nitrogen, and sulfur isotope ratios in human hair in South Korea

**DOI:** 10.1371/journal.pone.0256404

**Published:** 2021-08-20

**Authors:** Han-Seul Lee, Ji-Yu Shim, Woo-Jin Shin, Seung-Hyun Choi, Yeon-Sik Bong, Kwang-Sik Lee

**Affiliations:** 1 Research Center for Geochronology and Isotope Analysis, Korea Basic Science Institute, Cheongju-si, Chungbuk, Republic of Korea; 2 Graduate School of Analytical Science and Technology, Chungnam National University, Daejeon, Republic of Korea; 3 Center for Research Equipment, Korea Basic Science Institute, Cheongju-si, Chungbuk, Republic of Korea; Senckenberg Gesellschaft fur Naturforschung, GERMANY

## Abstract

Dietary homogenization has progressed worldwide due to westernization and the globalization of food production systems. We investigated dietary heterogeneity in South Korea by examining the spatial distribution of carbon (C), nitrogen (N), and sulfur (S) isotope ratios using 264 human hair samples. Overall, variation in isotope values was small, indicating low dietary heterogeneity. We detected differences in *δ*^13^C, *δ*^15^N, and *δ*^34^S values between administrative provinces and metropolitan cities; inter-regional differences were typically < 1 ‰. Values of *δ*^34^S were significantly lower in hair samples from inland regions relative to those from coastal locations, and a similar pattern was observed in *δ*^15^N values. Understanding geographic variation in *δ*^34^S and *δ*^15^N values in human hair is useful for provenancing humans in South Korea.

## Introduction

Stable isotope analyses of bio-elements, including carbon (C), hydrogen (H), nitrogen (N), oxygen (O), and sulfur (S), are commonly used to investigate the dietary protein sources consumed by both contemporary and past human populations [1–4]. Dietary intake patterns are recorded in body tissues such as hair, nail, bones, and teeth, which allow for the reconstruction of lifestyle attributes related to geographic origin and dietary habits [5–7]. In particular, human hair is a continually growing, readily sampled tissue that can serve as a longitudinal record of movement history [8–11]. In contrast to other body tissues, hair has no metabolic activity after being produced, and diet characteristics, region-of-origin, and recent travel history are thus stored in this tissue [12–14].

Over the past two decades, scientists have investigated the spatial distribution of bio-element stable isotopes in human tissues for forensic analyses [2, 10]. Among stable isotopes, H and O isotope ratios in human hair are closely related to drinking water source [5]. Regional-scale distributions of H and O isotope ratios in modern human hair were documented in the United States (USA) by Ehleringer et al. [5] and showed geographically predictable variations. Isoscapes, visualizations of spatial distributions of isotopic signatures, are a valuable tool for mapping heterogeneity against various environmental and geographic parameters and provide useful baseline information for provenance and forensic inquiries. Isoscape studies focused on H and O values in water and hair have been conducted worldwide (e.g., [5, 15]). Recently, Gautam et al. [16, 17] demonstrated that H and O isotope signatures in human hair can be used in provenance- and forensic-related activities, such as residential history and migration history in South Korea, based on a clear latitudinal gradient in isotope compositions. Their study demonstrated the predictive ability of H and O isoscapes to determine the regional origin of hair in South Korea.

Unlike H and O isotopes, the stable isotope ratios of C, N, and S are typically used for dietary reconstruction studies because their ratios in human tissues are entirely related to food consumption [2]. Therefore, stable isotopes of C, N, and S in human tissues can complement H and O isotope analyses [2]. Hülsemann et al. [18] provided an excellent global overview of C and N isotope patterns using approximately 4,000 individual human hair samples and reported significant differences in *δ*^13^C and *δ*^15^N values among countries or regions. Broadly, human dietary patterns have homogenized due to rapid globalization in food production and distribution processes, resulting in reduced differences in isotope values among regions [3]. However, dietary heterogeneity is still preserved among European countries [6], North American countries [2, 19], and some regions in Japan [3].

The global spatial distribution of *δ*^13^C values in human hair is strongly related to the amount of C4 plants in human diets [18, 20] and *δ*^15^N values are reflective of trophic level, and can thus indicate whether an individual is omnivorous or vegetarian if they consume only terrestrial foods [4]. Individuals who consume large amounts of fish and seafood are expected to have higher *δ*^15^N values than those who mainly eat terrestrial foods [21]. Generally, *δ*^34^S values are reflective of the consumption of foods from different geological regions. Specifically, marine-derived foods exhibit approximately +20 ‰ of *δ*^34^S values [2]. Thus, *δ*^34^S values can reflect both diet and geographic origin [19, 22, 23]. Factors potentially explaining differences in *δ*^34^S values include bedrock geochemistry [11, 22], atmospheric deposition of sea-spray salts on coastal soils and vegetation [19, 24], and the consumption of fish- or meat-derived proteins [2, 15]. Valenzuela et al. [2] demonstrated distinct geographic pattern in *δ*^34^S values in the USA using human hair samples, where inland values were much lower than those from coastal regions. These regional differences can then be applied in forensic provenancing studies [2, 18, 19].

Datasets of stable isotope values determined from human hair are highly biased to North America and Europe, with limited data available from other countries [18]. Minimal data are available from East Asia, with only limited information about *δ*^13^C and *δ*^15^N values in human hair available from South Korea. To our knowledge, no study has examined spatial variation and heterogeneity in C, N, and S isotopes in human hair at a national scale in South Korea.

The use of multi-isotopes from human tissues improves the predictive power of isoscapes [22]. Building from the recently published H and O isoscapes created using human hair samples from South Korea [16, 17], we investigated spatial distributions of *δ*^13^C, *δ*^15^N, and *δ*^34^S values in human hair samples to extend the applicability of isotopes for human provenancing using a multi-isotope approach. We further investigated dietary heterogeneity and characterized the dietary patterns of contemporary Koreans using C, N, and S isotopes. Finally, we compared isotope ratios from the Korean hair samples with those from other countries to characterize dietary protein sources in Korea in a global context.

## Materials and methods

### Ethical statement and data availability

This study was approved by the Bioethics Committee at the Korea Basic Science Institute (IRB number: 005). Hair samples were collected from trash bins with oral consent of the owners of barbershops. Only samples identified as male hair were collected from barbershops. Biological sex was the only donor information collected. Isotope data and sample locations are available in S1 Table and S2 Table.

### Sample collection and preparation

We collected 264 human scalp hair samples from trash bins at barbershops for men in South Korea. Hair samples were placed in individual plastic bags at the time of collection. To ensure that the isotopic signature of hair samples reflected the geographic location of sampling regions, hair samples were obtained from barbershops located in residential areas. We assumed that the collected hair samples were representative of men residing in the area around the collection site.

Prior to analysis, to remove lipids and surface contaminants, approximately 3 g of hair from a bundle of multiple hairs from the same individual were placed in a 100-mL beaker, washed using 0.5% Triton-X (Sigma Aldrich, St Louis, MO, USA), Milli-Q water (> 18 MΩ cm), and acetone (Honeywell Burdick & Jackson, Muskegon, MI, USA), and then rinsed twice with Milli-Q water. The amount of solvent used for each wash was sufficient to completely submerge the hair samples, and each step was treated in an ultrasonic bath for 2 min. Washed samples were then fully dried in a drying oven at 60°C overnight. Each dried sample was pulverized into a fine powder using a ball mill (MM400; Retsch, Haan, Germany). Homogenized samples were stored in vials prior to analysis. For the isotope analysis, 34 food samples were collected from supermarkets in metropolitan cities, subsequently, lipids were removed and samples were freeze-dried at −80°C for 72 h and then ground using a ceramic ball mill.

### Stable isotope analyses

C, N, and S isotope ratios were determined using an isotope ratio mass spectrometer (IRMS; Isoprime VisION, Isoprime Ltd., Manchester, UK) that was equipped with a vario PyroCube elemental analyzer (Elementar, Hesse, Germany) that included a combustion tube maintained at 1150°C and a U-shaped adsorption column to separate the generated CO_2_ gas; the analyzer was operated in continuous flow mode. For the C isotope ratio, approximately 0.1 mg of prepared hair sample was encapsulated in a tin container (3.5 × 1.5 mm; Elemental Microanalysis, Okehampton, UK). For the N and S isotope ratios, approximately 0.8 mg of prepared hair sample was encapsulated in tin capsules, respectively. The tin capsules were stored in a desiccator until analysis to avoid vapor absorption. The encapsulated sample was combusted at 1150°C in a combustion reactor and excess oxides were removed in a reduction reactor at 850°C. Thereafter, N_2_, CO_2_, and SO_2_ gases that had been generated were separated using chromatography and the isotope ratios were measured using the IRMS. Because of varying content of C, N, and S in hair samples, C, N, and S isotopes were analyzed separately.

Stable isotope ratios were reported using the standard delta notation (*δ*) relative to an international standard unit per mill (‰) as follows: *δ* (‰) = (R_sample_/R_reference_ − 1) × 1000, where R_sample_ and R_reference_ are the molar ratios of the heavy to light isotopes of the sample and standard, respectively, representing ^13^C/^12^C, ^15^N/^14^N, or ^34^S/^32^S. The resulting *δ*^13^C, *δ*^15^N, and *δ*^34^S values were reported against Vienna Pee Dee Belemnite (VPDB), atmospheric nitrogen (Air), and Vienna Canyon Diablo Troilite (VCDT), respectively. The analytical precision was within ± 0.1 ‰ for C, ± 0.2 ‰ for N, and ± 0.2 ‰ for S.

The following standard reference materials were used for the calibration of C, N, and S isotope ratios: NBS-22 (oil, *δ*^13^C_VPDB_ = −29.8 ‰), IAEA-CH-3 (cellulose, *δ*^13^C_VPDB_ = −24.73 ‰), IAEA-CH-6 (sucrose, *δ*^13^C_VPDB_ = −10.45 ‰), IAEA-600 (caffeine, *δ* ^13^C_VPDB_ = −27.77 ‰, *δ*^15^N_Air_ = +1.0 ‰), USGS-40 (l-glutamic acid, *δ* ^13^C_VPDB_ = −26.39 ‰, *δ*^15^N_Air_ = −4.52 ‰), USGS-42 (Tibetan human hair, *δ*^13^C_VPDB_ = −21.09 ‰, *δ*^15^N_Air_ = +8.05 ‰, *δ*^34^S_VCDT_ = +7.84 ‰), USGS-43 (Indian human hair, *δ*^13^C_VPDB_ = −21.28 ‰, *δ*^15^N_Air_ = +8.44 ‰, *δ*^34^S_VCDT_ = +10.46 ‰), IAEA-N-2 (ammonium sulfate, *δ*^15^N_Air_ = +20.3 ‰), IAEA-SO-5 (barium sulfate, *δ*^34^S_VCDT_ = +0.5 ‰), and NBS-127 (barium sulfate, *δ*^34^S_VCDT_ = +21.1 ‰).

### Geostatistical analyses

Normality was tested using the Shapiro-Wilk test. P-values were considered significant at the α = 0.05 level. We used Student’s t-tests to compare mean isotope values between two groups and used analysis of variance (ANOVA) to compare isotope values among provinces. Statistical significance was interpreted based on *p* < 0.05. These analyses were conducted using XLSTAT Pro software (Addinsoft, New York, NY, USA).

The spatial distributions of the *δ*^13^C, *δ*^15^N, and *δ*^34^S values obtained from the hair samples were visualized across South Korea (including the province of Jeju Island) using interpolation mapping. Maps were produced using ordinary kriging (OK), a common geostatistical interpolation method, and we did not consider the application of other interpolation methods (e.g., empirical Bayesian kriging, simple kriging). The OK was applied using ArcGIS version 10.8 (ESRI, Redlands, CA, USA). In addition, spatial autocorrelation (Moran’s index) was performed using the software to examine the spatial distribution pattern of human hair (clustered, dispersed, or random).

## Results and discussion

### C, N, and S isotope ratios in human hair and food

The range and mean values of C, N, and S isotope ratios obtained from hair samples collected from nine administrative provinces and seven metropolitan cities in South Korea are summarized in Table 1.

**Table 1:** Carbon, nitrogen and sulfur isotope values of human hair samples collected from nine administrative provinces (GG through JJ) and major Metropolitan Cities (MC) in South Korea.

Province	*δ*^13^C (‰)			*δ*^15^N (‰)			*δ*^34^S (‰)			n
	Min	Max	Mean ± SD	Min	Max	Mean ± SD	Min	Max	Mean ± SD	
**GG**	-20.2	-18.1	-19.1 ± 0.5^a^^b^	8.4	10.5	9.5 ± 0.4^c^^d^	5.7	8.2	7.0 ± 0.5^b^	27
**GW**	-20.2	-18.3	-19.2 ± 0.4^a^^b^	8.4	10.4	9.5 ± 0.4^c^^d^	3.4	9.7	7.1 ± 1.2^b^	29
**CN**	-20.1	-18.5	-19.3 ± 0.4^a^^b^^c^	8.5	10.6	9.7 ± 0.4^a^^b^^c^^d^	6.1	9.4	7.4 ± 0.7^a^^b^	27
**CB**	-20.4	-18.7	-19.5 ± 0.4^b^^c^	8.8	9.7	9.3 ± 0.3^d^	6.1	7.5	6.8 ± 0.3^b^	20
**GB**	-20.2	-18.2	-19.3 ± 0.5^a^^b^^c^	9.0	11.1	9.8 ± 0.5^a^^b^^c^	6.3	10.0	7.5 ± 0.9^a^^b^	36
**JB**	-20.9	-18.4	-19.6 ± 0.6^c^	8.6	10.3	9.6 ± 0.4^b^^c^^d^	6.1	8.2	7.4 ± 0.5^a^^b^	26
**JN**	-20.8	-18.1	-19.3 ± 0.6^a^^b^^c^	9.2	11.7	10.0 ± 0.6^a^	6.1	9.9	7.4 ± 0.8^a^^b^	29
**GN**	-20.5	-18.3	-19.1 ± 0.4^a^^b^	8.5	11.3	9.9 ± 0.5^a^^b^	6.1	9.4	7.5 ± 0.8^a^^b^	28
**JJ**	-19.5	-17.6	-18.9 ± 0.4^a^	9.0	10.3	9.6 ± 0.3^b^^c^^d^	6.2	8.8	7.8 ± 0.7^a^	25
**MC**	-20.2	-18.0	-19.0 ± 0.5^a^^b^	9.1	11.4	9.9 ± 0.5^a^^b^^c^	6.4	9.7	7.3 ± 0.8^a^^b^	17
**Total**	-20.9	-17.6	-19.2 ± 0.5	8.4	11.7	9.7 ± 0.5	3.4	10.0	7.3 ± 0.8	264

Provinces are abbreviated as: Gyeonggi (GG), Gangwon (GW), Chungbuk (CB), Chungnam (CN), Jeonbuk (JB), Jeonnam (JN), Gyeongbuk (GB), Gyeongnam (GN), and Jeju (JJ). MC includes Seoul, Incheon, Daejeon, Gwangju, Daegu, Ulsan and Busan metropolitan cities. The C, N and S isotope values for provinces were compared by ANOVA and Tukey’s HSD test at p = 0.05 level.

^a–abcd^ Different letters in each column indicate significant statistical differences (p < 0.05, Tukey’s HSD test).

Overall, the mean *δ*^13^C value among all hair samples was −19.2 ± 0.5 ‰ (range: −20.9 ‰ to −17.6 ‰). The mean *δ*^15^N value was +9.7 ± 0.5 ‰ (range: +8.4 to +11.7 ‰). The mean *δ*^34^S value was +7.3 ± 0.8 ‰ (range: +3.4 to +10.0 ‰). The *δ*^13^C, *δ*^15^N, and *δ*^34^S values obtained from food from local stores and supermarkets are shown in Table 2 [25–32].

**Table 2:** Carbon, nitrogen and sulfur isotope values of food samples collected from South Korea.

Sample	*δ*^13^C (‰) (mean ± SD)	n	*δ*^15^N (‰) (mean ± SD)	n	*δ*^34^S (‰) (mean ± SD)	n	Reference
**Chicken**	-18.6 ± 0.7	2	2.3 ± 0.7	2	1.3 ± 2.1	2	This study
**Egg**	-18.1 ± 0.3	4	4.0 ± 0.2	4	5.1 ± 0.3	4	This study
**Tofu**	-28.9 ± 0.2	2	1.7 ± 1.1	2	1.0 ± 0.4	2	This study
**Apple**	-26.6 ± 1.2	2	7.7 ± 0.1	2	N/A	N/A	This study
**Marine fish**	-19.7 ± 2.5	20	10.6 ± 3.3	20	17.8 ± 2.3	20	This study
**Sea weed**	-21.0 ± 4.7	4	4.8 ± 2.6	4	20.6 ± 0.1	4	This study
**Beef (USA)**	-13.1 ± 2.2	30	6.3 ± 0.7	10	N/A	N/A	[25, 26]
**Beef (AUS)**	-20.0 ± 4.7	31	7.3 ± 1.9	10	N/A	N/A	[25, 26]
**Beef (KOR)**	-17.0 ± 1.1	25	6.2 ± 0.4	23	N/A	N/A	[25, 26]
**Beef (NZ)**	-25.3 ± 1.7	25	5.8 ± 0.6	5	N/A	N/A	[25, 26]
**Beef (MX)**	-12.6 ± 1.0	3	7.0 ± 0.2	3	N/A	N/A	[25, 26]
**Pork**	-19.2 ± 0.9	611	3.6 ± 0.5	611	N/A	N/A	[27–29]
**Milk**	-22.3 ± 0.4	286	5.0 ± 0.3	286	5.9 ± 0.3	100	[30–32]
**Rice**	-27.5 ± 0.7	9	6.3 ± 0.3	9	2.1 ± 1.9	9	[33]
**Potato**	-26.6 ± 0.4	60	6.4 ± 0.6	60	3.2 ± 0.6	60	[34]
**Mushroom**	-24.4 ± 0.1	81	13.7 ± 0.7	81	10.0 ± 0.4	81	[35]
**Onion**	-25.9 ± 1.0	130	3.6 ± 2.8	130	-0.3 ± 2.3	130	[36]
**Garlic**	-26.2 ± 0.8	163	2.8 ± 3.3	163	-1.6 ± 2.1	163	[37]

N/A indicates “not analyzed”.

**Fig 1:**
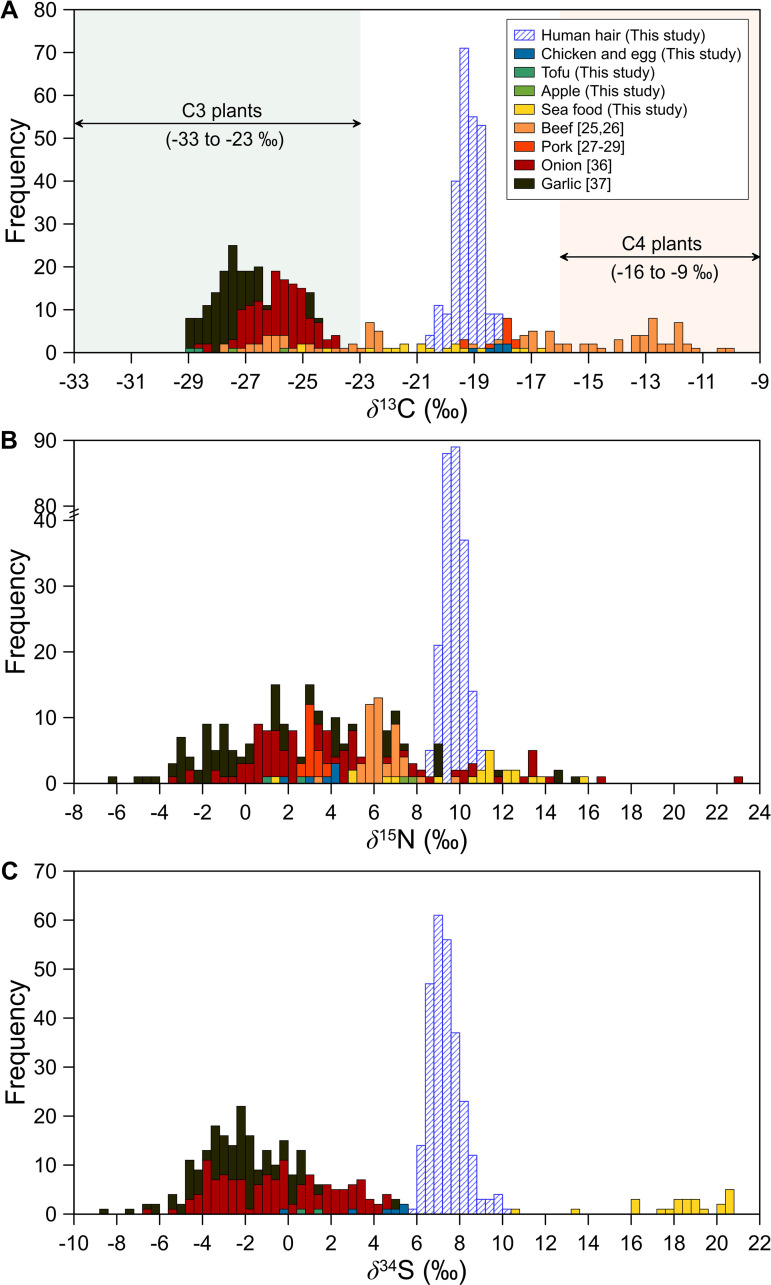
Frequency distributions of (A) *δ*^13^C, (B) *δ*^15^N, and (C) *δ*^34^S values in human hair and food samples collected across South Korea.

Frequency distributions of C, N, and S isotope ratios in both hair and food samples are shown in Fig 1. The *δ*^13^C values in food samples ranged from −29.1 ‰ to −9.9 ‰ (n = 545), indicative of both C3 and C4 plants. The majority of the food samples were reflective of C3 plants (Fig 1A). Generally, the results indicated that Koreans consume a wide variety of foods, inclusive of C3 and C4 plants [33–37], because the *δ*^13^C values from hair samples corresponded to the mid-range value between C3 and C4 plants. The typical *δ*^13^C ranges of C3 plants and C4 plants are -33 ‰ to -23 ‰ and -16 ‰ to -9 ‰, respectively [36]. According to the Korea Rural Economic Institute [38], rice, wheat, and corn accounted for 55.4%, 22.7%, and 18.9% of all grains consumed in South Korea, respectively, in 2003–2018. Rice and wheat are C3 plants, while corn is a C4 plant; thus, the *δ*^13^C values obtained from hair samples are at least partially reflective of the relative abundance of these foods in an individual’s diet.

Unlike *δ*^13^C values, the distribution of *δ*^15^N values differed significantly among food and hair samples (Fig 1B), where those from food samples were significantly lower. The *δ*^15^N values from food samples ranged from −6.2 ‰ to +16.8 ‰ (mean +4.3 ± 4.0 ‰, n = 481), with the exception of one sample greatly enriched in ^15^N (+23.0 ‰), and approximately 60% of the food samples occurred within the range of +2 to +8 ‰. Thus, the average *δ*^15^N value in human hair samples was +5.4 ‰ higher than that from food samples. An elevated *δ*^15^N value in hair is strongly related to animal protein consumption and is reflective of individual dietary patterns. The supply of animal-based protein (approximately 56% of the total dietary protein) is significantly higher than plant-based protein (approximately 44% of total protein) in South Korea [39]. Thus, animal protein consumption is likely responsible for high *δ*^15^N values in Korean hair samples.

The *δ*^34^S values from both food and hair samples showed a similar pattern to that of *δ*^15^N values, and seafood samples with relatively higher *δ*^34^S values were more clearly separated from human hair and other food samples (Fig 1C). Generally, high consumption of marine-based and animal proteins is reflected by high *δ*^15^N and *δ*^34^S values in hair [2], and high *δ*^34^S values are associated with high intake of marine-based protein or the sea spray effect (Fig 1C; [19, 40]). We visualized the spatial distributions of *δ*^15^N and *δ*^34^S values of rice (unpublished data) and garlic [41] samples collected across South Korea, and little or no differences were observed in *δ*^15^N and *δ*^34^S values between coastal and inland crops. Such spatial distribution may indicate little or no sea-spray effects around coastal areas in the mainland. However, Jeju Island (JJ), located in the southern part of the Korean Peninsula is the administrative province most affected by sea-spray. Jeju Island samples were collected close to the coast, so local foods may have contained particularly high amounts of marine aerosols. Garlic samples collected from Jeju Island had slightly higher *δ*^34^S values than those collected from the mainland, indicating a weak sea-spray effect. Meanwhile, *δ*^15^N values of pork meat samples produced on Jeju Island did not differ from those produced on the mainland (*p* = 0.23, n = 16) [29]. No *δ*^34^S data for animal meats such as pork, beef and chicken are currently available for South Korea. Therefore, given the *δ*^15^N and *δ* ^34^S data of agro-products available for South Korea, high values of *δ*^34^S and *δ*^15^N in coastal human hairs from the mainland can primarily be interpreted as a result of fish and seafood consumption [19].

**Fig 2:**
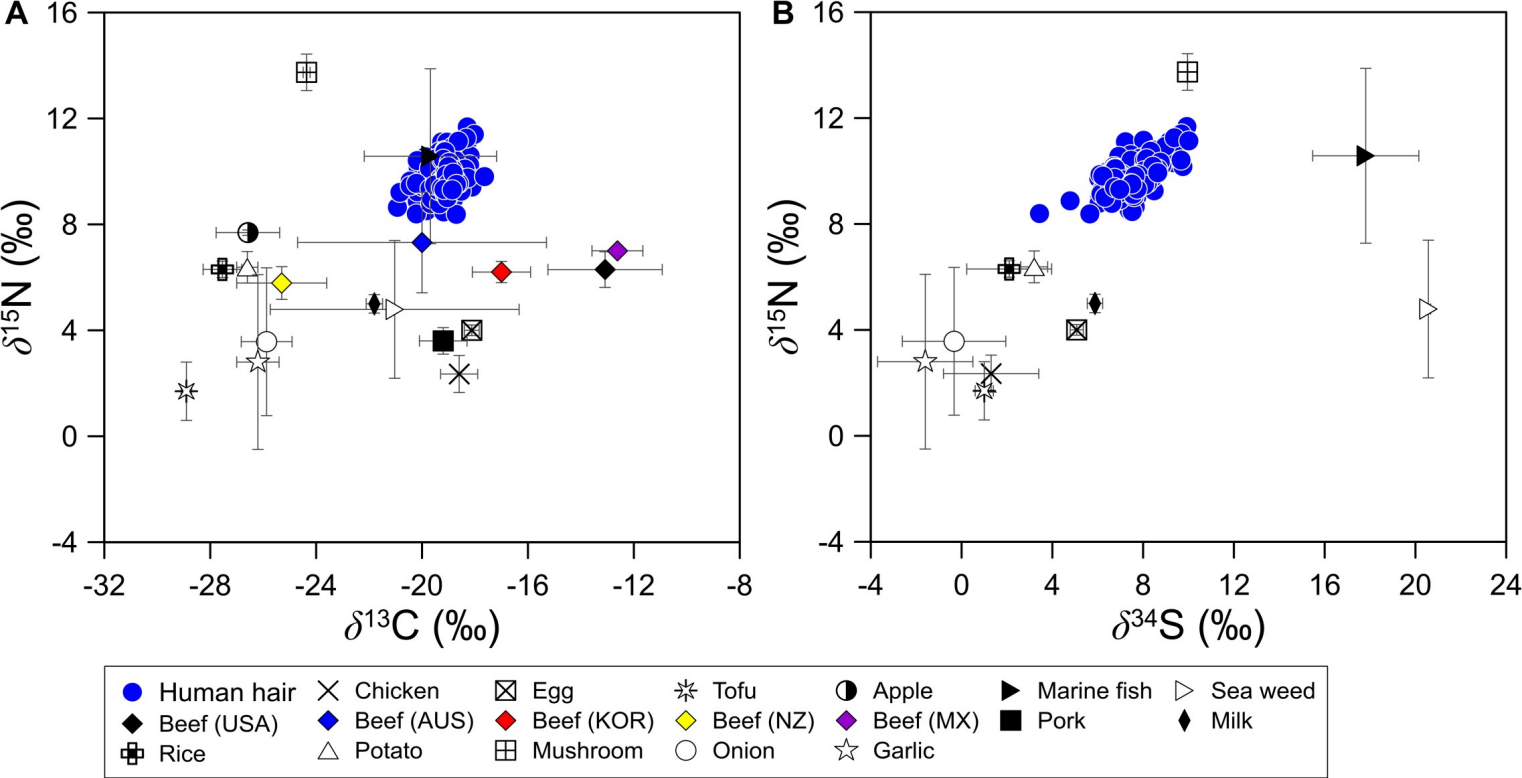
Relationships between (A) *δ*^13^C and *δ*^15^N, and (B) *δ*^15^N and *δ*^34^S values obtained from human hair and food samples collected across South Korea.

The results of the isotope analyses of hair and food samples are shown in Fig 2. Broadly, the *δ*^13^C and *δ*^15^N values from food samples were scattered across a wide range, whereas those from hair samples were confined to a narrow range. This may be because hair reflects the average isotope values of various food samples.

Samples of C3 plants (apples, onions, and garlic) and tofu made from soybean (C3 plant) had low *δ*^13^C values (Table 2); *δ*^13^C values in livestock products were higher and those from milk samples were similar to C3 plants. The *δ*^13^C values in marine fish and seaweeds ranged from −24.2 ‰ to −14.3 ‰ and −25.1 ‰ to −16.7 ‰, respectively. The *δ*^15^N values of marine fish were high relative to C3 plants, livestock products, and seaweeds, excluding mushroom samples, which have exceptionally high *δ*^15^N values due to the use of organic fertilizers such as animal manure [35]. In South Korea, *A*. *bisporus* mushrooms are usually cultivated with a compost medium in a greenhouse system, and not with soil-based cultivation [35]. The high *δ*^15^N value observed for *A*. *bisporus* may be associated with the *δ*^15^N values of the animal manure in the medium used for mushroom cultivation, which could reflect the N isotope fractionation that occurs between the medium and the mushroom fruit body during mushroom cultivation [35]. In addition, *δ*^15^N values for hair samples exhibited a similar range to those of marine fish, which were markedly higher than values in other samples. This finding indicates that humans are top-level predators in food chains, and marine fishes, which are predators in more complex food chains, can exhibit *δ*^15^N values similar to human hair.

Isotope ratios in livestock are reflective of their diets. Australian and New Zealand cattle are typically fed C3 plants, resulting in low *δ*^13^C values, whereas cattle in the USA and Mexico are typically fed corn, resulting in high *δ*^13^C values. In Korea, C3 and C4 plants are used to feed cattle; thus, *δ*^13^C values are intermediate to beef from the other continents [25, 26]. In our study, beef had a *δ*^15^N value that was approximately +3 ‰ higher than that of pork or chicken (Fig 2A) and the *δ*^34^S values in fish and seaweed were much higher than those in C3 plants and livestock products.

### Regional homogeneity and localization of isotope ratios

**Fig 3:**
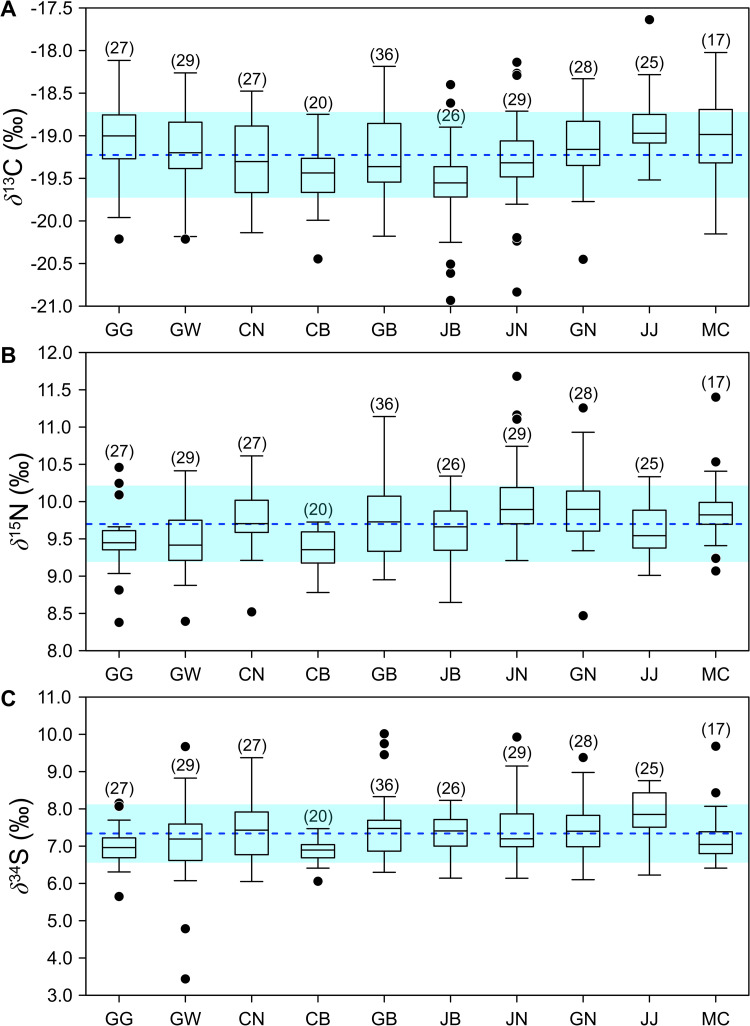
Boxplots showing (A) *δ*^13^C, (B) *δ*^15^N, and (C) *δ*^34^S values in human hair samples collected from nine administrative provinces (GG through JJ) and major metropolitan cities (MC) in Korea. Provinces are abbreviated as: Gyeonggi (GG), Gangwon (GW), Chungbuk (CB), Chungnam (CN), Jeonbuk (JB), Jeonnam (JN), Gyeongbuk (GB), Gyeongnam (GN), and Jeju (JJ). Seven metropolitan cities (hatched areas) are also shown. Dashed lines represent the mean isotope values of all samples. Colored bars represent the standard deviations of isotope values for all samples.

We found no significant difference in the intake rate of animal and marine foods between metropolitan cities and administrative provinces in South Korea. On average, animal and marine food intake is reportedly only 1.2% higher in metropolitan cities in South Korea [37]. Data regarding the intake rates of animal and marine products were not available for all administrative provinces considered here. Although we expected minimal variation in *δ*^13^C, *δ*^15^N, and *δ*^34^S values in human hair from cities and provinces, we found significant differences among some provinces (Table 1) and small standard deviations within provinces (± 0.6 ‰ or less for both *δ*^13^C and *δ*^15^N). Comparisons of isotope values for all provinces and cities are provided in Fig 3.

Among all provinces, mean *δ*^13^C values were slightly higher in Jeju Island samples (−18.9 ± 0.4 ‰) and lowest in Jeonbuk (−19.6 ± 0.6 ‰). Mean *δ*^15^N was highest in Jeonnam (+10.0 ± 0.6 ‰) and lowest in Chungbuk (+9.3 ± 0.3 ‰), the only inland province that is not in contact with the sea. The mean *δ*^34^S value was highest in Jeju Island (+7.8 ± 0.7 ‰) and lowest in Chungbuk (+6.8 ± 0.3 ‰). Intra-region variation in *δ*^34^S was highest in Gangwon (± 1.2 ‰). Overall, the variation in *δ*^34^S values was low and did not deviate significantly from the mean.

Dependency on imported food, rather than food sovereignty, is increasing annually in South Korea [39], which would be expected to decrease regional differences in isotope ratios. Dietary patterns also tend to change and homogenize with industrialization and economic development. Consequently, food products in Korean grocery stores reflect global supermarkets due to changes in consumption patterns driven by the expansion of food markets and services. This trend toward globalization is likely driving the relatively homogenous pattern we observed in hair isotope values (Fig 3). According to Korea’s Food Balance Sheet of 2018 [39], Korea produced only 21.9% of the grain its population consumed, with the remaining 78.1% imported from the USA, Australia, and Brazil. Excluding rice, of which Korea produced 82.5% of its own amount consumed, the bulk of the major agricultural products on today’s shelves are imported. In addition, the self-sufficiency rate of animal food items, excluding eggs, has sharply declined.

### Comparison of isotope values in hair among countries

**Fig 4:**
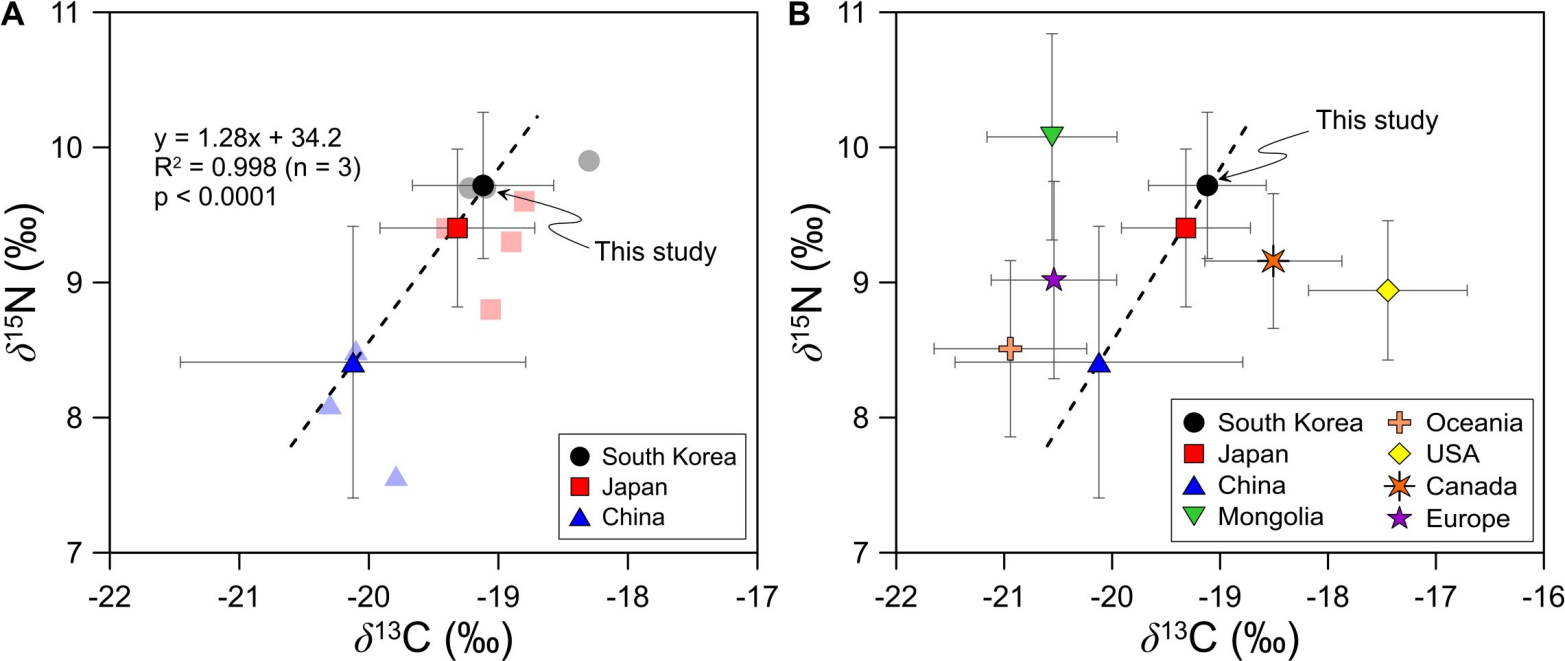
Plots of *δ*^13^C and *δ*^15^N values of human hair. (A) *δ*^13^C and *δ*^15^N values found in human hair in South Korea, China, and Japan and (B) the same data including observations from Mongolia, Oceania, USA, Canada and Europe for comparison. In the figure on the left, symbols without error bars represent the average values of individual studies in each country (circle: South Korea, square: Japan, triangle: China). Data sources: South Korea [3, 42], Japan [3, 42–44], China [18, 44, 45], Mongolia [3, 42], Oceania [18, 44, 46–48], USA [2, 4, 6, 18, 42, 49, 50], Canada [18, 19, 44], Europe [6, 8, 18, 44, 51, 52].

To characterize the dietary patterns of contemporary Koreans on a global scale, we compared the isotope ratios obtained from the hair samples with those from other countries (Fig 4). We focused on comparisons with findings from East Asia (South Korea, Japan, and China) (Fig 4).

The *δ*^13^C and *δ*^15^N values in Korean hair obtained in this study are similar to those reported previously [3, 42]; thus, it is assumed that isotopic variation in hair samples is not large. In particular, *δ*^13^C and *δ*^15^N values in Korean hair were similar to those in Japanese hair [3, 42–45]. Given the *δ*^13^C values, the consumption rates of C3 and C4 plants are similar between Japan and Korea (Fig 4A).

For comparison, the USA, which mainly consumes C4 plants reflected by high *δ*^13^C values [18], and the European Union (EU), which mainly consumes C3 plants reflected by low *δ*^13^C values [4], are also shown (Fig 4B). Canada [18, 19, 44] and Oceania [18, 44, 46–48] are also illustrated (Fig 4B). The *δ*^13^C values of Korean hair were intermediate to those of the USA (+ Canada) [2, 4, 6, 18, 19, 42, 44, 49, 50] and EU (+ Oceania) [6, 8, 18, 44, 51, 52], suggesting proportional relationships with the consumption of C3 and C4 plants and seafood. Based on both *δ*^13^C and *δ*^15^N values, male Koreans eat both C3 and C4 plants and are omnivorous.

According to the Food and Agriculture Organization of the United Nations [53], Korean people consumed significantly less fish and animal protein than Japanese people on a gram per day basis decades ago. However, this gap has narrowed since 2004 and fish and animal protein intake between these two countries has been similar in recent years (S1 Fig; [53]). Thus, *δ*^15^N values obtained from contemporary Korean and Japanese hair samples should be similar, as was observed here.

The *δ*^13^C and *δ*^15^N values were higher in Korean hair relative to Chinese samples (Fig 4A). This may be because Chinese people are more dependent on plant protein intake and thus have lower *δ*^15^N values than Korean and Japanese people (S1 Fig; [53]). The low *δ*^15^N values of Chinese hair are also due to consumption of legumes or poultry with soy protein [22]. We found a positive correlation in *δ*^13^C and *δ*^15^N values among China, Japan, and South Korea (*δ*^15^N = 1.27*δ^13^C + 33.98, R^2^ = 0.996, *p* < 0.05). However, data from Mongolia deviated considerably from the correlation line (Fig 4B). The *δ*^13^C values of Mongolian hair were significantly lower than those in Japanese and Korean hair, whereas *δ*^15^N values were similar or slightly elevated [3, 45]. This may be reflective of a distinct lifestyle and dietary habit among Mongolians, who prefer a diet with a high intake of animal protein and little fish protein (S2 Fig; [53–55]).

The range of variation of *δ*^13^C and *δ*^15^N values in Korean and Japanese hair samples were smaller than those of Chinese hair samples. This is likely because foods consumed by Korean and Japanese people are characteristic of a global supermarket, with more homogenous isotope compositions [3]. By contrast, subtle differences in food types in China and Mongolia can be identified by city [45], indicating that diets including local foods have been maintained.

### Spatial distributions of C, N, and S isotopes

**Fig 5:**
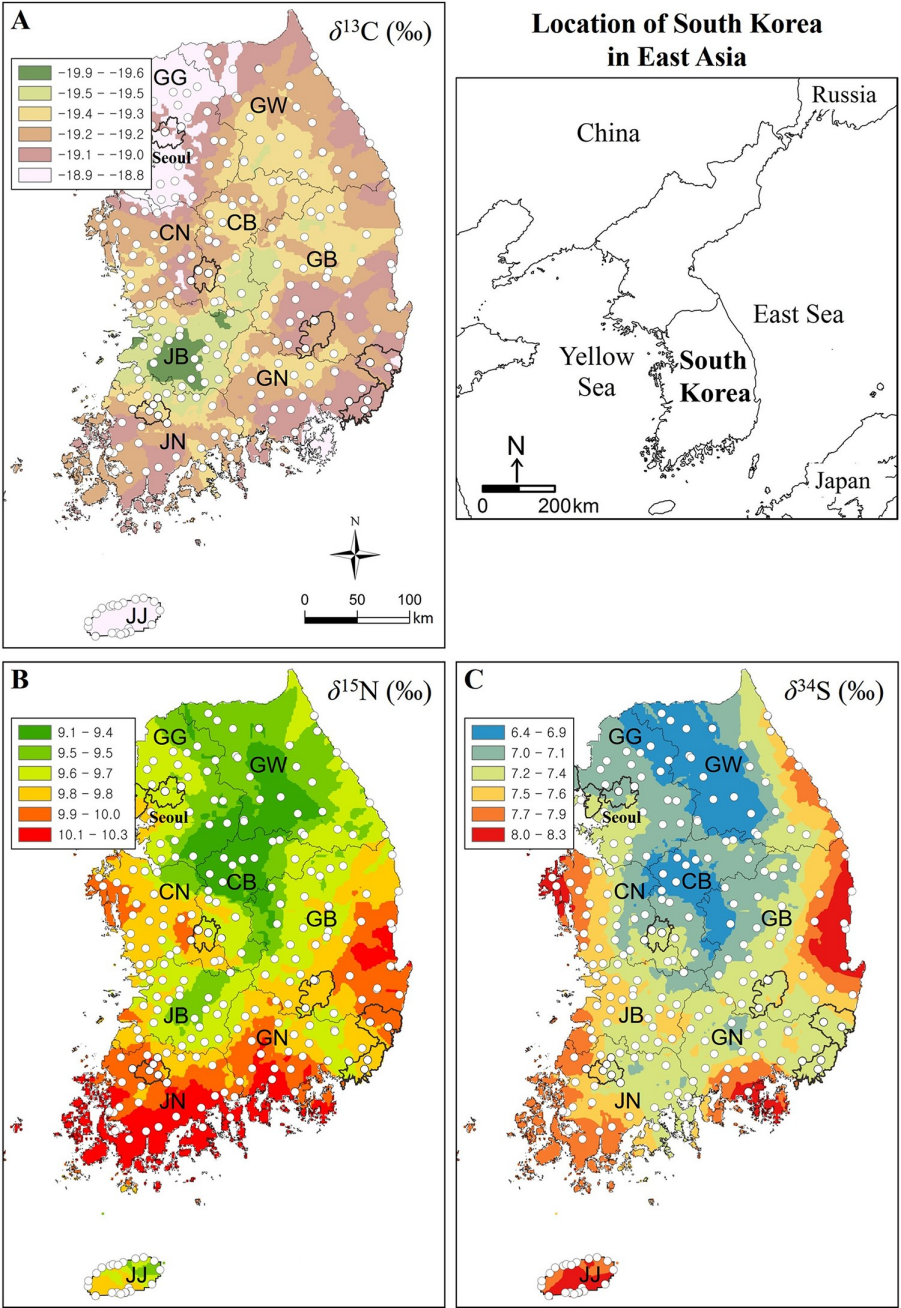
Isoscapes of (A) *δ*^13^C, (B) *δ*^15^N, and (C) *δ*^34^S in South Korea, with sampling locations indicated by open circles and seven metropolitan cities shown by bold lines. Maps were produced using ordinary kriging (OK), a common geostatistical interpolation method using ArcGIS version 10.8 (ESRI, Redlands, CA, USA). The method used in this classification is natural break (Jenks). The administrative boundaries of nine administrative provinces are shown. Provinces are abbreviated as: Gyeonggi (GG), Gangwon (GW), Chungbuk (CB), Chungnam (CN), Jeonbuk (JB), Jeonnam (JN), Gyeongbuk (GB), Gyeongnam (GN), and Jeju (JJ).

The spatial distributions of *δ*^13^C, *δ*^15^N, and *δ*^34^S values in human hair samples for South Korea are shown in Fig 5. A weak but interesting pattern was observed in the C isoscape (Fig 5A). In Seoul, the largest metropolitan city in South Korea, and its surrounding areas, *δ*^13^C values were slightly higher than in other regions. This was a consistent, albeit weak, pattern that was reflected in other metropolitan cities. Jeju Island, which sees high international traffic, also had higher *δ*^13^C values. Globalization has had the most rapid advancement in metropolitan cities and on Jeju Island within South Korea. Westernization and globalization tend to relate to increased consumption of foods containing corn [2, 4]. Corn, a C4 plant, relates to high *δ*^13^C values [56, 57]. Consequently, consuming corn-fed animals also increases the *δ*^13^C values in human hair. Excluding the areas mentioned above, we saw little effect of geographic and environmental factors on *δ*^13^C values (Fig 5A).

By contrast, there was a distinct geographic pattern in *δ*^15^N values. Specifically, N isotope values are markedly low in the northern to western inland and high along the southern coast (including the southeastern coast), that is, distinctively low in mountainous areas and high in agricultural areas (Fig 5B). Organic fertilizers such as animal manures (chicken and farmyard manures; *δ*^15^N = +14.7 ± 7.7 ‰, n = 30) are primarily used in agricultural lands in South Korea [36, 58]; thus, the spatial variation of *δ*^15^N values seems to be related to the consumption of food produced in these areas. Generally, *δ*^15^N values are also determined by the consumption of fish and meat [1, 56, 57, 59, 60] and the high values along the coastal regions are unsurprising, as marine-derived proteins have high *δ*^15^N values [61, 62].

**Fig 6:**
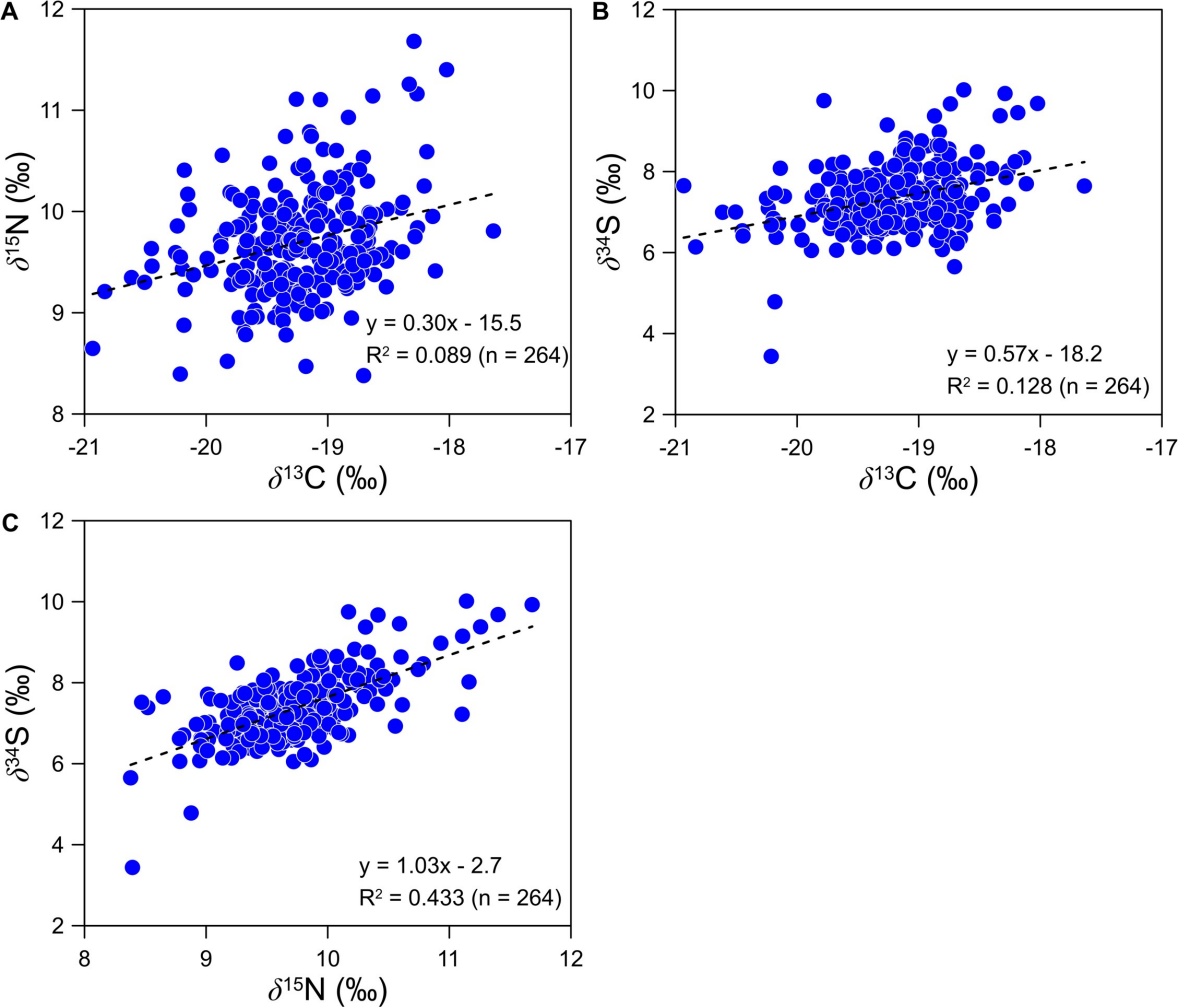
Correlation plots showing the relationships between (A) *δ*^13^C and *δ*^15^N, (B) *δ*^13^C and *δ*^34^S, and (C) *δ*^15^N and *δ*^34^S values in human hair samples collected from barbershops across South Korea. A stronger correlation between *δ*^15^N and *δ*^34^S values was observed.

Finally, *δ*^34^S values of human scalp hair in South Korea showed a distinct geographic pattern similar to that of *δ*^15^N values (Fig 5C), significantly lower in inland relative to coastal regions. Studies have shown that proximity to the sea can have a substantial influence on N and S isotope values, with higher *δ*^15^N and *δ*^34^S values in people who consume a large amount of marine products [22, 24]. A weak correlation was detected between *δ*^13^C and *δ*^15^N values (R^2^ = 0.089, *p* < 0.001, n = 264, Fig 6) and between *δ*^13^C and *δ*^34^S values (R^2^ = 0.128, *p* < 0.001, n = 264) from human hair samples. However, a stronger correlation was observed between *δ*^15^N and *δ*^34^S values (R^2^ = 0.443, *p* < 0.001, n = 264).

Spatial autocorrelation was employed to evaluate the distributions of *δ*^15^N and *δ*^34^S values for human hair. For the *δ*^15^N and *δ*^34^S values, the Moran’s I index was 0.356 (z = 1.328, *p* = 0.184) and 0.141 (z = 0.538, *p* = 0.591), respectively. Data lacking significant autocorrelations suggest that human hairs in terms of *δ*^15^N and *δ*^34^S values are not spatially autocorrelated but are instead randomly dispersed. This observation can be explained by the possibility that the isotopic compositions of human hair could reflect both geographical and topographical features. Likewise, agricultural practices more extensively occurred in the lower elevation areas than in mountainous areas. The distribution of lowlands and highlands is not consistent with distance from marine environments. Thus, the isotopic value in each sampling region would be compensated or integrated, statistically resulting in the absence of spatial autocorrelation. From this perspective, the randomly dispersed- spatial distribution would also be attributed to westernization and globalization. Nonetheless, the interpolated map illustrates a distribution pattern that reflects land use and distance from marine environments.

Given the patterns we observed among all isotope values, we conclude that dietary patterns have become homogenized in Korea due to westernization and globalization, irrespective of region, but that dietary heterogeneity still exists in some provinces. Our results indicate that C, N, and S isotopic signatures in human hair are strongly correlated with the food consumed by individuals. These regional differences in mean *δ*^13^C, *δ*^15^N, and *δ*^34^S values are useful for provenancing studies and allow us a more holistic view of geographic patterns following the publication of H and O isotope data.

## Conclusions

We collected 264 human scalp hair samples across South Korea to investigate dietary heterogeneity and spatial variation in C, N, and S isotopes at a national scale. To our knowledge, this is the first national-scale study assessing C, N, and S isotope ratios in human hair in South Korea. The mean values of *δ*^13^C, *δ*^15^N, and *δ*^34^S in hair samples were −19.2 ± 0.5 ‰, +9.7 ± 0.5 ‰, and +7.3 ± 0.8 ‰, respectively. The inter-regional difference in C isotope ratios was < 0.5 ‰, indicating dietary homogeneity due to rapid westernization and globalization. Nevertheless, regional dietary heterogeneity still exists in some areas in South Korea.

Spatial products like isoscapes are valuable tools because they allow us to visualize spatial heterogeneity in isotopes given environmental parameters, such as distance to sea. We found that *δ*^34^S values in human hair were greatly influenced by proximity to marine environments in South Korea, with higher values in coastal relative to inland regions. Interestingly, the N isoscape showed a similar pattern to that of the S isoscape. The consistency between the two illustrates the potential of isoscapes to act as a proxy for food source in South Korea, and can provide useful data for forensics and migration tracking. Further, understanding C, N, and S isoscapes in South Korea contributes to the growing literature on the identification of residential history using isotopic analyses of human hair.

## Supporting information

S1 Table *δ*^13^C, *δ*^15^N, and *δ*^34^S values of human hair samples (n = 264) collected from South Korea. (XLSX)

S2 Table *δ*^13^C, *δ*^15^N, and *δ*^34^S values of food samples (n = 34) collected from metropolitan cities supermarkets in South Korea. (XLSX)

S1 Fig (A) Fish and (B) meat per capita supply for East Asian countries since 1961.
(TIF)

S2 Fig Fish and animal contribution to protein supply for East Asian countries since 1961: (A) fish/total proteins, (B) animal/total proteins and (C) (fish+animal)/total proteins.
(TIF)
