# Supplementary figures and images for: Dietary homogenization and spatial distributions of carbon, nitrogen, and sulfur isotope ratios in human hair in South Korea
Source: PLoS One. 2021 Aug 20;16(8):e0256404. doi: 10.1371/journal.pone.0256404 (PMC8378694; doi:10.1371/journal.pone.0256404)

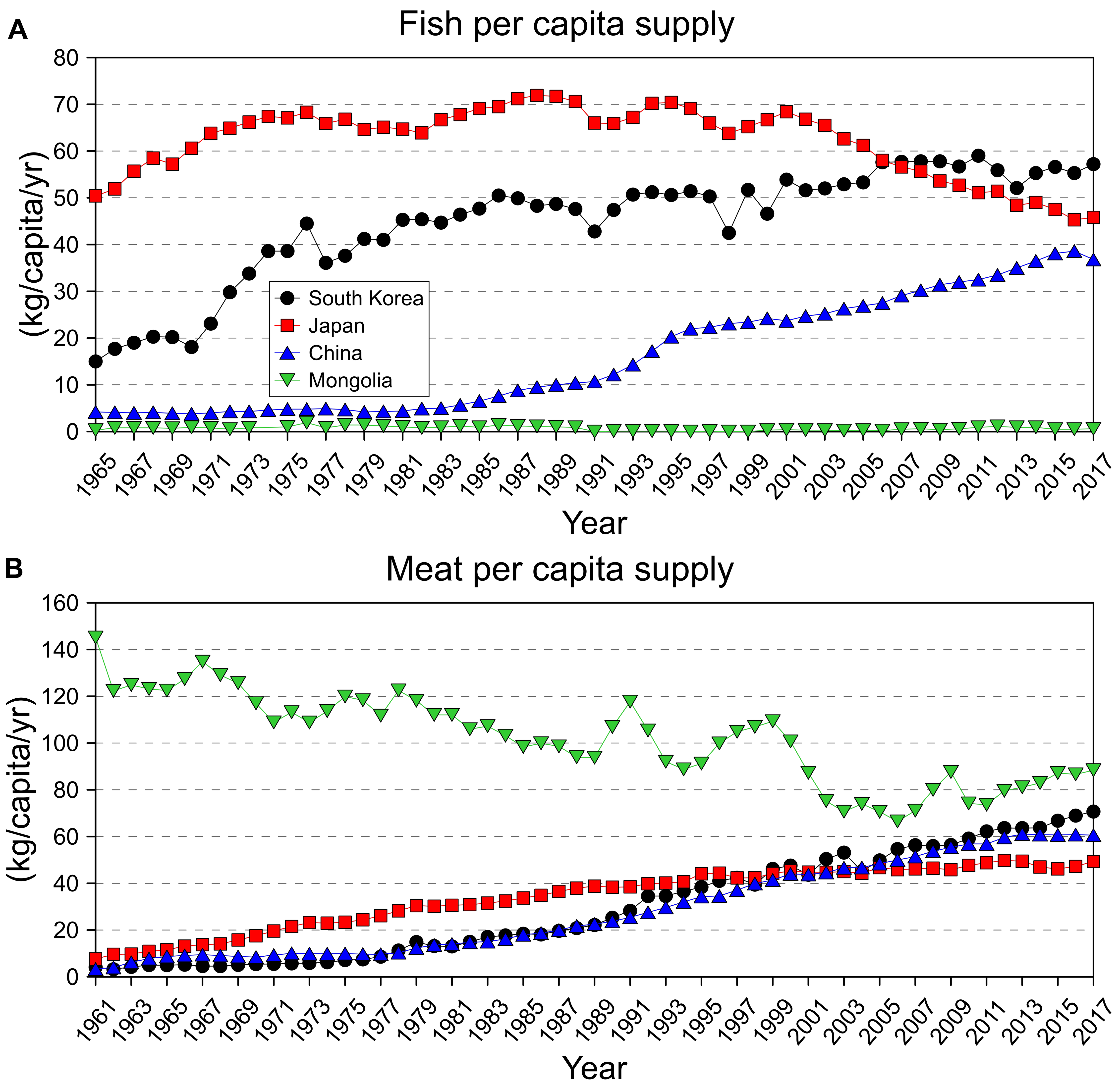

Supplement: S1 Fig — (A) Fish and (B) meat per capita supply for East Asian countries since 1961. (TIF) [file pone.0256404.s003.tif]

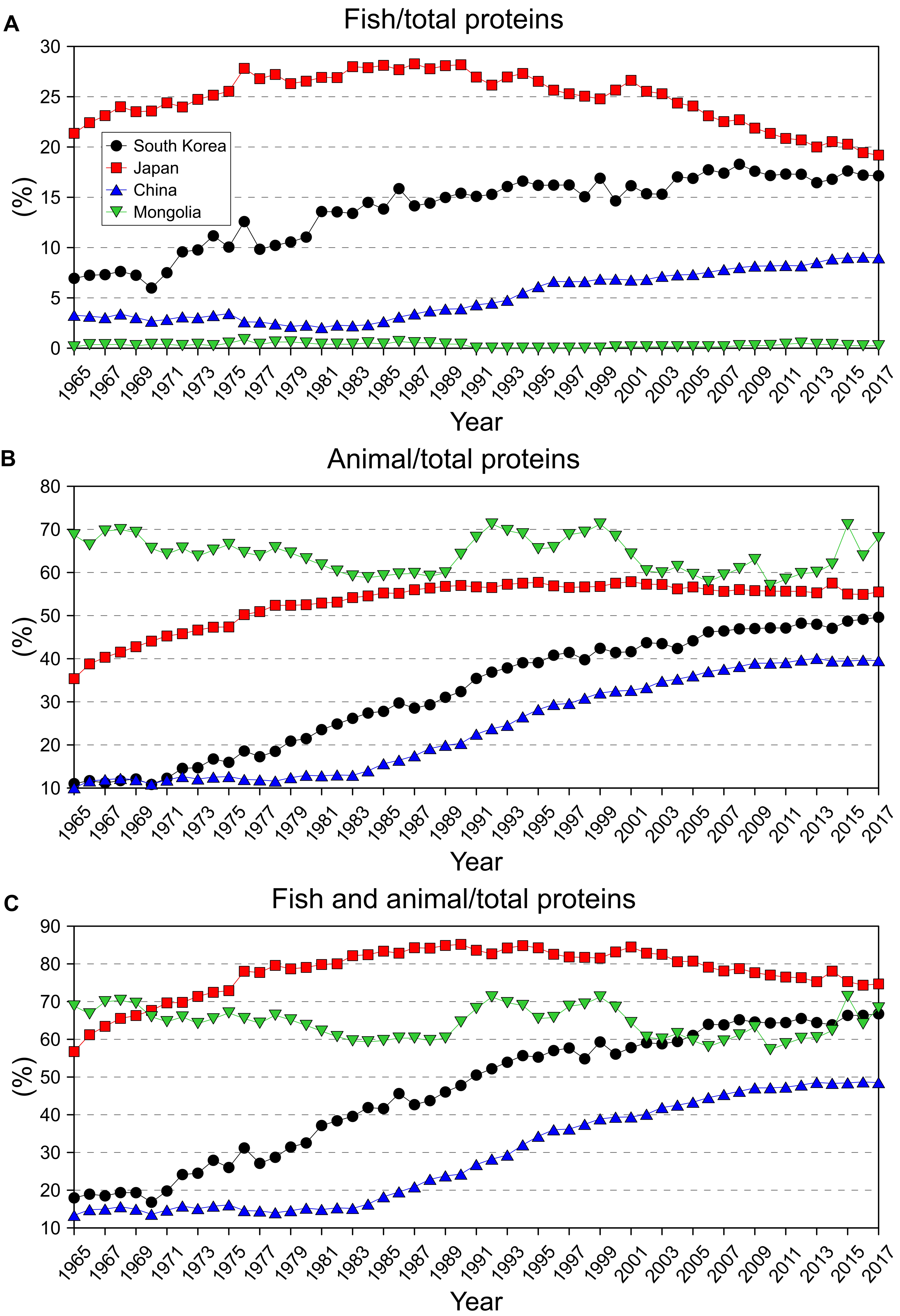

Supplement: S2 Fig — Fish and animal contribution to protein supply for East Asian countries since 1961: (A) fish/total proteins, (B) animal/total proteins and (C) (fish+animal)/total proteins. (TIF) [file pone.0256404.s004.tif]
